# A retrospective epidemiological analysis of maxillofacial fractures at a tertiary referral hospital in istanbul: a seven-year study of 1,757 patients

**DOI:** 10.1186/s40902-024-00447-4

**Published:** 2024-11-06

**Authors:** Orhan Asya, Yavuz Gündoğdu, Sefa İncaz, Ömer Tarık Kavak, Javahir Mammadli, Sefa Özcan, Celal Emre Çavlan, Ali Cemal Yumuşakhuylu

**Affiliations:** 1 Faculty of Medicine, Department of Otorhinolaryngology, Marmara University, Pendik Training and Research Hospital, Fevzi Çakmak, Muhsin Yazıcıoğlu Street, Istanbul, 34899 Turkey; 2 Private Otorhinolaryngology Practice, Metropol Istanbul Residence, Block A, Floor 44, No: 570, Istanbul, 34758 Turkey; 3 VM Medical Park Maltepe Hospital , Cevizli, Bagdat Street, Istanbul, 34899 Turkey

**Keywords:** Maxillofacial fractures, Trauma, Etiology, Facial fractures, Epidemiology

## Abstract

**Background:**

The aim of the study was to evaluate the etiology, incidence, demographics, and characteristics of maxillofacial fractures treated at a university hospital over a seven-year period.

**Methods:**

We performed a retrospective analysis of 1,757 patients with maxillofacial fractures who were referred to our department between May 2012 and March 2019. The patients’ demographic and clinical characteristics were noted, as well as the fracture type, location, and etiology. The treatment modalities were also analyzed.

**Results:**

A total of 2,173 maxillofacial fractures were seen in 1,757 patients. The male to female ratio was 3.9:1, and the mean patient age was 31.89 ± 17.70 years (range: 0–95 years). Maxillofacial injuries were most prevalent in the 19–28 years age group (23.9% of cases), with a general increase in injuries observed between 2013 and 2018 across all age groups. The most common etiological factor was assault (29.1%), followed by falls (26%). In male patients, assault was reported as the main cause, while in female patients, falls were identified as the main cause. The nasal bone was the most common site of fracture, followed by the maxilla. The average time from admission to surgery was 2.8 days, with local anesthesia being the most frequent surgical intervention. The average time from admission to surgery was 2.8 ± 2.5 days (range: 0–20 days), with surgeries performed under local anesthesia being more frequent than those carried out under general anesthesia. Among the surgical interventions, the most common general anesthesia technique for fracture reduction was open reduction and internal fixation with plates and screws. Plate exposure, wound-site infection, and temporomandibular joint ankylosis were the major complications encountered in the study population.

**Conclusion:**

The study reveals significant variability in maxillofacial fractures based on gender, age, and etiology. Assault emerged as the leading cause of these fractures, followed by falls and road traffic accidents. Men were affected by maxillofacial trauma four times more often than women, with the highest incidence occurring in the 19–28 years age group. Nasal fractures were the most frequently observed (78.7%), while condylar-subcondylar process fractures were the most common type of mandibular fracture. Given these findings, a targeted, lifelong prevention strategy focused on high-risk groups could significantly reduce the burden of maxillofacial trauma.

## Background

Maxillofacial trauma accounts for approximately 5–33% of all severe trauma cases [1–3] and is a significant health concern because the maxillofacial region is involved in critical functions, such as vision, hearing, olfaction, respiration, mastication, and speech [4]. Maxillofacial trauma can occur in isolation and in conjunction with other severe traumas. It can lead to physiological dysfunction as well as an increase in psychological morbidity due to subsequent alterations in the patient’s aesthetic appearance.

The incidence and causes of facial fractures vary significantly from country to country (and even within countries) and depend on various factors, including geographical location, culture, lifestyle, population density, and socioeconomic trends [5, 6]. Common causes of maxillofacial fractures are road traffic accidents (RTAs), assaults, falls, and sports and work injuries. While RTAs are reportedly the primary cause of facial fractures in developing countries, assaults and falls have been found to be the most common cause of facial fractures in developed countries [7]. There is now a decreasing trend in RTA-related facial trauma in several developed countries due to changes in road safety legislation, such as the introduction of mandatory seat belt use, speed limits, and drink driving penalties [7].

It is critical to acquire deep knowledge and a thorough understanding of the incidence and causes of facial injuries to identify the trauma burden, devise necessary precautions, and formulate countermeasures. Hence, evaluating the causes, frequency, and severity of maxillofacial trauma plays an important role in establishing effective treatment and prevention measures.

Based on our experience, there is a lack of information on the causes and other epidemiological factors of maxillofacial injury in Turkey, as well as on the treatment strategies implemented. There is currently no research available or published on the epidemiology of maxillofacial injuries in Istanbul, which is home to 18.33% of the Turkish population, including etiologies, related factors, or treatment strategies. Thus, the purpose of this study was to analyze the demographics, causes, and types of maxillofacial fractures followed at the Otorhinolaryngology—Head and Neck Surgery Department of a tertiary medical center in Istanbul, the most populated city in Turkey, over a seven-year period. We also examined the treatment modalities utilized and the management of patients with facial trauma. The findings of this study can be used to guide the funding of prevention-oriented public health programs.

## Methods

### Study design

This retrospective descriptive study was conducted in the Otorhinolaryngology and Head and Neck Surgery Department of a tertiary medical center. It involved the examination of the medical and radiological records of patients with maxillofacial injury who were referred to the department by Emergency Department (ED) staff between 2012 and 2019. The study was approved by the tertiary medical faculty Ethics Committee for clinical research (#09.2019.492). Respect for the privacy and rights of the participants was maintained throughout the study. Because the study was designed retrospectively, informed consent was not obtained from the patients.

Our hospital serves as a major trauma center on the Anatolian side of Istanbul. In our hospital, both the Otorhinolaryngology Department and the Plastic Surgery Department manage patients with maxillofacial trauma. As a major trauma center on the Anatolian side of Istanbul, our hospital manages maxillofacial trauma patients through both the Otorhinolaryngology and Plastic Surgery departments. According to the agreement between the departments, all patients attending the ED with maxillofacial injury are referred to the Department of Otorhinolaryngology for one month and to the Plastic Surgery Department for the next month on a rotating basis. Thus, the patients included in this study correspond to approximately half of the patients with maxillofacial injury who were admitted to the hospital during the study period.

### Participants and data collection

Of the 1,864 patients with maxillofacial trauma referred to our department during the study period, 1,757 were included in this study; 107 patients were excluded due to incomplete data. Demographic and clinical characteristics of the patients, the etiology of the injury, the location and type of fracture, and the treatment modality were noted. Clinical records were selected from patients referred to our department from the ED for the treatment of maxillofacial trauma between 1 May 2012 and 31 March 2019. Patients were included if they had been diagnosed with either displaced or non-displaced bony fractures in the maxillofacial region and if their records contained complete demographic and clinical data necessary for our study objectives. Patients with incomplete data, those with isolated soft tissue injuries without osseous pathology, and those who discharged themselves against medical advice or refused hospital treatment were excluded from the study. All included patients underwent computed tomography (CT) scanning of the maxillofacial region. Table 1 summarizes the data extracted from the clinical records for analysis.

**Table 1:** Variables extracted from clinical records for analysis

**Demographic data (**Age, Gender)
**Clinical data (**Etiology of injury, Location of fracture *)
**Treatment modalities (**Conservative management, Intervention with Local anesthesia, Surgical intervention with General anesthesia)
**Time between admission and surgery** Duration between hospital admission and surgical intervention
**Postoperative complications (**Plate exposure, wound-site infection, and temporomandibular joint (TMJ) ankylosis)

The patients’ facial injuries were noted according to their anatomical location (i.e., nasal, mandibular, zygomatic arch, zygomatic complex, maxillary, orbital, walls of the frontal sinus, and Le Fort fractures). Le Fort fractures were subclassified as Le Fort type I, II, or III, and orbital fractures were subclassified according to the involved wall. Mandibular fractures included fractures of the condyle-subcondyle, ramus, coronoid, angle, corpus, and symphysis/parasymphysis.

Participants were categorized by gender (female, male) and age (0–7, 8–18, 19–28, 29–38, 39–48, 49–58, and ≥ 59 years). The cause of the injury (in-vehicle traffic accident, pedestrian accident, motorcycle accident, fall, assault, sport injury, etc.) and the type and location of the fracture were noted. The treatment modalities were also noted, such as conservative management, closed reduction, open reduction and fixation with plates and screws, orbital titanium mesh, and elevation of the zygomatic bone with a temporal or oral approach.

## Statistical analysis

All data were categorical and presented as frequency (n) and percentage (%). Data were grouped based on key variables, including age groups, gender, mandibular fracture subunit, and the etiology of maxillofacial fractures. The distribution of categorical variables was compared using the Chi-Square (χ2) test, which was also employed for post hoc analysis of subgroups with two or more categories. To assess differences in age between male and female patients, an independent samples t-test was used, as the age data were normally distributed. The normality of age distribution was evaluated using the Shapiro–Wilk test, and homogeneity of variance was assessed with Levene’s test. Statistical analyses were performed using SPSS for Mac (SPSS Inc., Chicago, IL), with results considered statistically significant at a p-value of less than 0.05. All data are categorical and presented as frequency (n) and percentage (%). Groups were compared using the χ^2^ test. The χ^2^ test was also used for the post hoc analysis of subgroups with two or more categories. Statistical analyses were performed using SPSS for Mac (SPSS Inc., Chicago, IL). Results with a *p*-value of less than 0.05 were considered statistically significant.

## Results

### Demographic information of the patients

Of the 1,757 patients with 2,173 maxillofacial injuries included in this study, 1,395 (79.4%) were male and 362 (20.6%) were female, resulting in a male-to-female ratio of approximately 3.9:1. The male-to-female ratio was observed to change between 2012 and 2019. The ratio was 7.1:1 in 2012, when there were 34 patients. Between 2013 and 2019, the ratio ranged from a minimum of 3:1 to a maximum of 4.7:1. There was no statistically significant difference in these ratios between 2013 and 2019 (p = 0.885). The mean patient age was 31.89 ± 17.70 years (range: 0–95 years). No statistically significant difference was observed between the mean ages of the male and female patients (male: 31.34 years, range: 0–93 years; female: 33.99 years, range: 0–95 years; P > 0.05).

All participants were classified according to their age, and the 19–28 years group was found to contain the most maxillofacial injuries, accounting for 23.9% of the cumulative distribution over seven years. The 0–7 years group was found to contain the least maxillofacial injuries. There was a trend of increasing injuries with age from the 0–7 years group to the 8–18 and 19–28 years groups. A decreasing trend in the distribution of injuries by age was observed in the 29–38, 39–48, 49–58, and ≥ 59 years groups, as shown in Fig. 2 and Table 2. Between 2013 and 2018, the number of patients with maxillofacial injuries increased significantly across all age groups. Figure 2 also includes the general population pyramids of Turkey for 2012 and 2019 to provide a visual comparison with the patient age distribution.

**Table 2:** Etiology of maxillofacial fractures among age groups

	**Age groups *(n)***	0–7	8–18	19–28	29–38	39–48	49–58	59 ≥	**Total *(n)***
**Etiology**	Motor vehicle accident	14	45	76	47	38	28	20	268
	Pedestrian accident	1	1	2	2	2	0	1	9
	Motorcycle accident	0	2	5	4	0	1	0	12
	Assault	1	78	137	152	89	41	13	511
	Work accident	0	5	14	12	10	5	2	48
	Sports accident	0	1	2	0	0	0	0	3
	Fall	57	111	76	55	50	39	68	456
	Other	1	25	18	25	5	5	14	93
	Unknown	14	77	90	83	43	23	27	357
	**Total**	88	345	420	380	237	142	145	1757

*Abbreviation*: *n* number of patients

## Yearly distribution of maxillofacial fractures

**Fig. 1:**
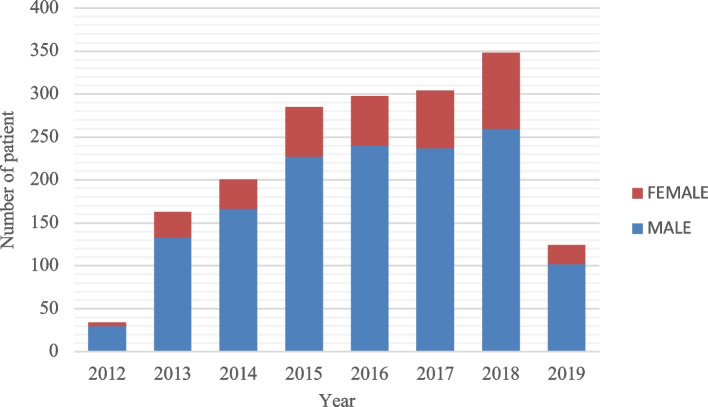
Annual distribution of the patients by gender**.** Annual distribution of maxillofacial trauma patients by gender from 2012 to 2019

Throughout the study period, a general increase in the number of male patients was noted, with the most significant increases observed in 2017 (*n* = 237) and 2018 (*n* = 260). Similarly, an increase was observed in the number of female patients, although the extent of the increase was not as great as that in the male population (Fig. 1).

## Etiology

The most common etiological factor associated with maxillofacial injury was found to be assault (29.1%, *n* = 511), followed by falls (26%, *n* = 456). In male patients, assault was reported as the main cause of maxillofacial injuries in 462 cases (33.1%), while in female patients, falls were identified as the main cause in 146 cases (40.3%), as shown in Table 3.

**Table 3:** Etiology of maxillofacial fractures among males and females

		**Male**		**Female**		**Total**		***P* value**
		*n*	*%*	*n*	*%*	*n*	*%*	
**Etiology**	Motor vehicle accident	212	15.2	56	15.4	268	15.3	0.988
	Pedestrian accident	5	0.4	4	1.1	9	0.5	0.920
	Motorcycle accident	12	0.8	0	0	12	0.7	0.920
	Assault	462	33.1	49	13.5	511	29.1	0.000^**^
	Work accident	45	3.2	3	0.8	48	2.7	0.620
	Sports accident	3	0.2	0	0	3	0.2	0.997
	Fall	310	22.2	146	40.3	456	26	0.000^**^
	Other	69	4.9	24	6.6	93	5.3	0.990
	Unknown	277	19.8	80	22.1	357	20.3	0.980
	Total	1395	100	362	100	1757	100	0.988

*Abbreviation*: *n* number of patients*; %* percentages

Chi-Square and Chi-Square post hoc analysis with Bonferroni correction (**p < 0.001)

In male patients, falls accounted for 310 (26%) cases, unknown causes for 277 (19.9%) cases, and in-vehicle traffic accidents for 212 (15.2%) cases. In female patients, the cause was unknown in 80 (22.1%) cases, and assault was the cause in 49 (13.5%) cases. The least common cause of injury among the male patients was sports accidents (*n* = 3, 0.8%), and among the female patients, it was work accidents (*n* = 3, 0.8%). Motorcycle- and sports-related injuries were not observed in any of the female patients over the seven-year period (Table 3).

χ^2^ statistics were used to examine the relationship between etiology and gender. A significant relationship was found between etiology and gender at the 5% significance level. The results of the χ^2^ post hoc analysis with Bonferroni correction showed that the etiology variable, categorized as patient gender, differed particularly in the categories of assault and falls, as shown in Table 3.

## Anatomical localization and type of maxillofacial fractures and treatment

The 1,757 patients presented with 2,173 maxillofacial fractures, or 1.23 fractures per patient (2,173 fractures /1,757 patients). The nasal region was the most common fracture location, and isolated nasal fractures or nasal fractures accompanying other fractures were detected in 1,384 (78.7%) patients. The most common underlying etiology of the recorded nasal fractures was assault (30.4%), and the second most common cause was falls (25.5%).

Among the patients diagnosed with nasal fractures, reduction of the fractured bone under local anesthesia (LA) was performed in 813 (58.9%) cases, reduction of the fractured bone under general anesthesia (GA) was performed in 29 (2.1%) cases, and the remaining 539 (39%) received conservative treatment.

## Mandibular fractures

A total of 117 patients with 155 mandibular fractures were identified; 90 had isolated mandibular fractures, and 27 had fractures associated with other facial injuries. The most common fracture sites in the mandible were the condylar-subcondylar process (*n* = 57, 36.8%), parasymphysis-symphysis (*n* = 43, 27.7%), corpus (*n* = 17, 11%), coronoid (*n* = 13, 8.4%), angle (*n* = 13, 8.4%), and ramus (*n* = 12, 7.7%), as shown in Table 4. The most common underlying etiology of these fractures was falls (*n* = 45, 38.5%), and the second most common cause was assault (*n* = 26, 22.2%).

**Table 4:** Anatomical occurance of mandibular fractures according to etiology

	**Etiology *(n)***	**Fall**	**Assault**	**RTA**	**Unknown**	**Other**	**Work accident**	**Total *(n)***
**Subunit**	Condyle-subcondyle	26	12	9	8	1	1	57
	Symphysis -Parasymphysis	20	9	5	4	5	0	43
	Corpus	4	2	2	5	4	0	17
	Angulus	2	7	0	4	0	0	13
	Coronoid	5	2	4	1	1	0	13
	Ramus	4	4	3	0	1	0	12
	Total	61	36	23	22	12	1	155

*Abbreviation*: *RTA* Road traffic accident; *n* number of patients

Motor vehicle accident, pedestrian accident and motorcycle accident were calculated under RTA

## Orbital fractures

There were 196 (9%) patients with 256 orbital fractures (37 with isolated fractures and 159 with fractures in combination with other facial injuries). The most common sites of fracture were the lateral wall (*n* = 67, 26.2%), floor (*n* = 61, 23.8%), inferior wall (*n* = 50, 19.5%), superior wall (*n* = 40, 15.6%), and medial wall (*n* = 38, 14.8%). The two most common causes of orbital fractures were assault (*n* = 47, 24%) and motor vehicle accidents (*n* = 47, 24%).

## Zygomatic arch fractures

Zygomatic arch fractures were observed in 121 patients (5.6%). The most common cause of zygomatic arch fractures was assault (*n* = 39, 32.2%), followed by falls (*n* = 32, 26.5%).

## Zygomatic complex fractures

Zygomatic complex fractures were observed in 63 patients (2.9%). The most common cause of zygomatic complex fractures was motor vehicle accidents (*n* = 21, 33.3%), and the second most common cause was assault (*n* = 16, 25.4%).

## Maxillary fractures

Maxillary fractures were observed in 228 patients (10.5%), either in isolation or in combination with fractures in other areas. The most common cause of maxillary fractures was assault (*n* = 55, 24.1%), and the second most common causes were motor vehicle accidents (*n* = 53, 23.3%) and falls (*n* = 53, 23.3%) at equal proportions.

## Frontal sinus anterior wall fractures

Frontal sinus anterior wall fractures were observed in 59 patients (2.7%), either in isolation or in combination with other areas. Isolated frontal sinus anterior wall fractures were observed in 15 patients (0.9%). The most common cause of the observed frontal sinus anterior wall fractures was assault (*n* = 13, 22%), and the second most common cause was falls (*n* = 12, 20.3%).

## Time between admission and surgery

The time between admission and surgical intervention ranged from 0 to 20 days. Among the patients who were treated via surgical repair with open reduction and internal fixation with plates and screws, only five patients underwent surgery on the day of admission, and the average time from admission to surgery was 2.8 ± 2.5 days (range: 0–20 days).

## Management and type of surgical intervention

Our analysis of the collected treatment modality data indicated that the most common type of intervention was intervention with LA (*n* = 845, 48.1%), followed by conservative treatment or monitoring (*n* = 768, 43.7%), and surgical intervention with GA (*n* = 130, 7.3%). Some patients declined surgery despite a need for it (*n* = 45, 2.6%). In other cases, surgery was not considered due to a general deterioration in the patient’s health (*n* = 14, 0.9%).

The most frequently performed procedures in patients who underwent surgical intervention with GA were open reduction and internal fixation with plates and screws, with or without intermaxillary fixation.

## Complications

We could only document major complications, namely plate exposure, wound-site infection, and temporomandibular joint (TMJ) ankylosis. Overall, 10 patients experienced these complications; there were six cases of plate exposure, three cases of postoperative wound-site infection, and one case of TMJ ankylosis.

Complications associated with maxillofacial fracture surgery include hemorrhage, nerve injury, infections, plate exposure, defective bone healing, and loss of or damage to teeth or bone [8]. We only observed plate exposure, wound-site infection, and TMJ related symptoms. Overall, 10 patients experienced these complications; there were six cases of plate exposure, three cases of postoperative wound-site infection, and one case of TMJ related symptoms.

## Discussion

There are many factors that may significantly impact the results of epidemiological studies, such as population density, geographic region, economic status, and cultural differences. Similarly, sociodemographic variables may influence the etiology, incidence, clinical presentation, and treatment strategies of maxillofacial fractures [9]. Hence, the epidemiological features of maxillofacial fractures vary across—and even within—countries [10].

Istanbul, which constitutes 18.33% of Turkey's population and has around 15 million residents, is bifurcated into two regions: the Anatolian and European sides. The area around our hospital is a rather underdeveloped part of Istanbul, characterized by low socioeconomic factors, inadequate educational attainment, and a high incidence of criminal activity. Despite our hospital's proximity to a relatively underdeveloped area of Istanbul concerning sociocultural factors, it receives referral patients from districs on the Anatolian side and less frequently also from the European side. As the preeminent maxillofacial institution on the Anatolian side, the data from our hospital is anticipated to reflect the totality of Istanbul. But to assess the assaults regarding their substantial contribution to the etiology, it is essential to acquire current district-based crime statistics for Istanbul and retrospectively identify the regions where the patients reside.

Previous studies from various regions of Turkey have reported similar male-to-female ratios and mean ages in maxillofacial trauma cases as observed in our study [11–14]. However, while RTAs are typically the leading cause in other regions in Turkey and also developing countries [11, 12, 15–17], our study found assault was the leading known cause of maxillofacial fractures (29.1%), followed by falls (26%) and RTAs (16.5%) similar to developed countries. Although factors such as age and gender remain consistent, the difference in etiology highlights the need for multicenter studies across Turkey to gain a more comprehensive understanding of the demographic and clinical characteristics of maxillofacial trauma nationwide.

**Fig. 2:**
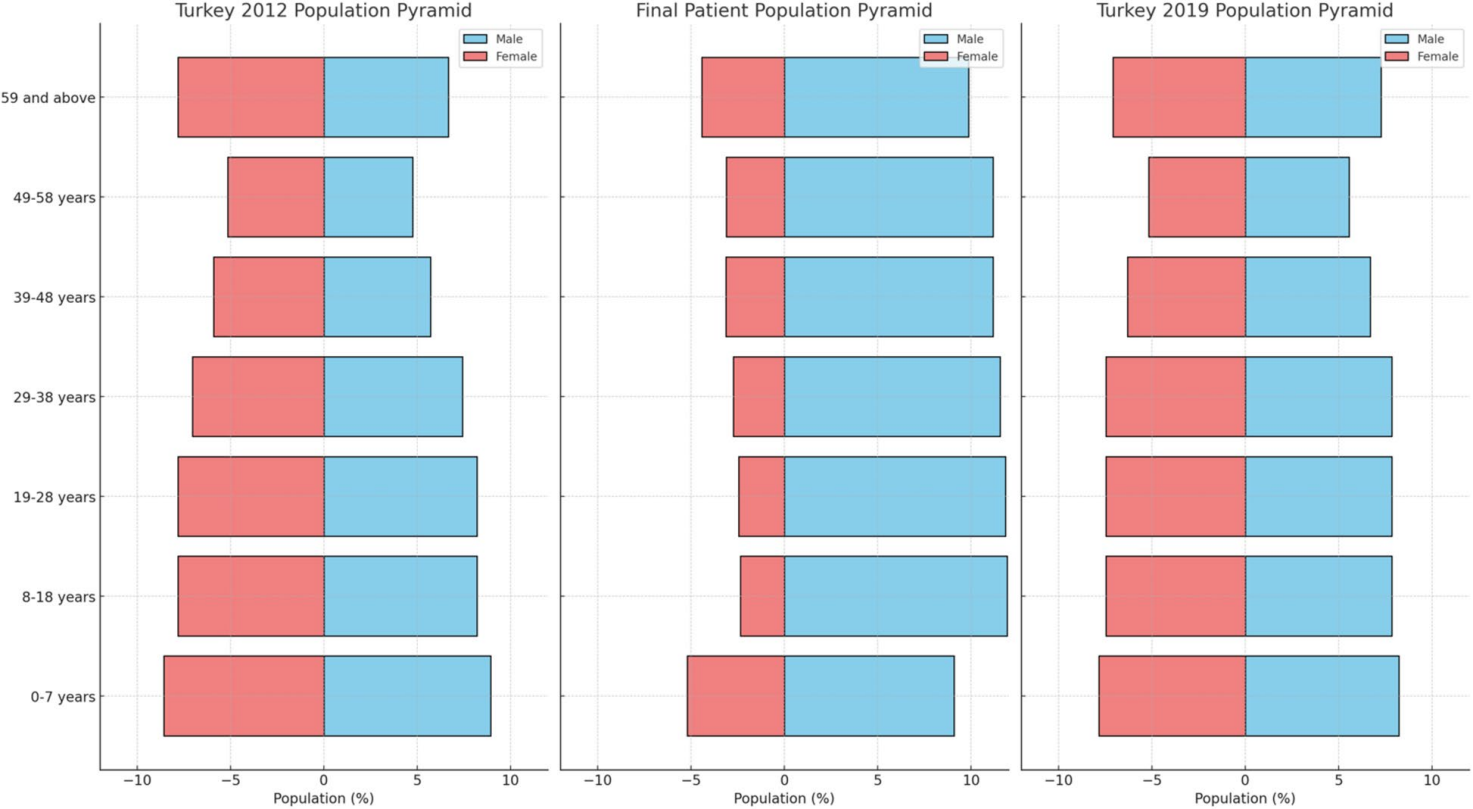
Comparison of Age and Gender Distribution Between Maxillofacial Trauma Patients and Turkey's General Population. The left panel shows the age and gender distribution of Turkey's general population in 2012, while the middle panel represents the distribution of the maxillofacial trauma patients treated between 2012 and 2019. The right panel illustrates the age and gender distribution of Turkey's general population in 2019. Data for Turkey's general population were sourced from the Turkish Statistical Institute (TÜİK) for 2012 and 2019

The comparison between patient distribution in our study and Turkey’s 2012 and 2019 population pyramids reveals a noticeable mismatch, as shown in Fig. 2. This discrepancy is likely due to the small sample size of 1,757 patients, which is insufficient to represent the national population accurately. Additionally, maxillofacial trauma predominantly affects young adults, which skews the age distribution compared to the general population. For more accurate results, future studies should include larger sample sizes or use national databases to better reflect both condition-specific and overall population demographics.

Men experience maxillofacial trauma significantly more than women all over the world and in all regions [18, 19]. While the male-to-female patient ratio is relatively high in underdeveloped and developing countries, it is lower and approaches 1:1 in developed countries. However, even in developed countries, men experience maxillofacial trauma almost twice as much as women. The male-to-female patient ratio is relatively high in underdeveloped and developing countries, while in developed countries, men experience maxillofacial trauma almost twice as often as women. In the literature, the male-to-female patient ratio ranges from 2:1 to 9.6:1 [5–7, 17, 20]. In the present study, the ratio was 3.9:1. In our study, the overall ratio was 3.9:1, which contrasts with findings from neighboring Middle Eastern countries, where ratios between 4.5:1 and 11:1 have been reported [21–23]. Excluding the statistics from 2012, when the ratio peaked at 7.5:1 due to a small sample size (34 patients), the annual male-to-female ratios observed in our study fluctuated between 4.7:1 and 3:1, with no statistically significant variation over time. The gender distribution found in our study is closer to that reported in urbanized European countries [24, 25]. This suggests that the greater involvement of women in society in Turkey, compared to other Middle Eastern countries, may increase their risk of experiencing trauma. In the European Maxillofacial Trauma (EURMAT) project [7], a multicenter prospective study including data from 13 hospitals across Europe, a mean male-to-female patient ratio of 3.6:1 was documented, which is similar to the ratio found in our study. However, in the EURMAT project, the ratio varied widely across the different centers and countries, with the maximum value encountered in Kiev (9.4:1) and the minimum value in Amsterdam (2.2:1) [7].

The etiology is an important epidemiological factor of maxillofacial fractures that directly affects the treatment modality, incidence, and clinical presentation. In the present study, assault was the leading known cause of maxillofacial fractures (29.1%), followed by falls (26%) and RTAs (16.5%). Studies have shown that in developing countries, the most common etiology of maxillofacial trauma is RTAs [15–17], while in developed countries, assault and interpersonal violence are the primary causes [7, 26]. Our findings reflect those of the EURMAT project, with the two most common etiologies of maxillofacial fractures being assault and falls. The region where our hospital is located is a relatively underdeveloped area of Istanbul in terms of sociocultural factors, the education level is low, and it is an area where criminal incidents frequently occur. These factors may have contributed to the occurrence of interpersonal and household violence and an increase in the number of assault cases during the study period.

In the present study, falls constituted the second most common etiology of maxillofacial fractures. Falls were found to be the most common cause of trauma in the 0–7, 8–18, and ≥ 59 years groups. Young children may experience falls due to a lack of movement certainty and coordination, which can prevent them from adequately protecting themselves against impact [27, 28]. Our data showed that in the 8–18 years group, falls were followed by assaults. The high incidence of assaults observed among school-age children is significant**.** Schools should increase education on this topic, parents must be made aware, and safety must be ensured. In accordance with the findings of the EURMAT project, falls were the most common etiology among the elderly patients (≥ 59 years group) in our study. Various factors may contribute to falls in this age group, including sensory impairment, neuromuscular disorders, unsteady gait, dementia, acute illness, and postural hypotension. In the EURMAT project, falls were the most common etiology across all centers in patients aged > 40 years [7]. This was attributed to the increasing age of the European population**.** Various steps should be taken to reduce the incidence of falls among the geriatric population, such as conducting home safety inspections, changing medications known to affect balance, providing gait training, and improving treatable sensory impairments through educational programs [29].

Comprehending the epidemiology of maxillofacial fractures and associated injuries is essential for formulating effective preventative programs, enhancing patient care, and judiciously allocating resources [30]. The occurrence and severity of maxillofacial fractures in adults and children can be markedly diminished by the implementation of seat restraints [31]. Nevertheless, adherence, especially among young males—who are the most vulnerable—tends to be inadequate [32]. Effective interventions encompass reducing speed limits, enforcing seatbelt compliance, and promoting helmet usage with chin shields to safeguard the mandible, the bone most frequently fractured in RTAs [33]. Moreover, adults responsible for supervising youngsters should instruct them on the significance of safe practices, including the use of helmets [34]. Supplementary strategies to reduce maxillofacial injuries from RTAs in developing nations include the installation of traffic cameras, radar systems, airbags, enhanced road design (prioritizing one-way streets), segregation of traffic kinds, safer pathways for pedestrians and cyclists, and the introduction of speed bumps [33]**.**

Geriatric patients face a heightened risk of future injuries following an initial event. Preventive interventions must encompass chronic diseases and functional limitations, including medication assessments, gait retraining, and environmental adjustments [35, 36]. Physical activity is the most efficacious strategy for fall prevention in elderly patients, and the ideal strategy is to combine it with other measures [37]**.**

Substance abuse exacerbates increasing incidences of violent trauma. Programs aimed at preventing alcohol and drug misuse might diminish motor vehicle accidents and injuries associated with assault, resulting in health and economic advantages. Legislative actions, such increasing the legal drinking age and enforcing stronger drunk driving regulations, may contribute to a decrease in alcohol-related facial fractures[38]**.**

Preventing osteoporosis is essential for overall health and may potentially influence maxillofacial injuries. Modifications in lifestyle, such as abstaining from smoking and limiting excessive use of alcohol and caffeine, in conjunction with regular exercise and adequate nutrition, can aid in the prevention of osteoporosis. Two categories of agents are available for its prevention and treatment: anti-resorptive and anabolic medicines[39]**.**

We could not determine the etiology in 20.3% (male: 19.9%; female: 22%) of the patients, which is a high proportion of the cases. Since this was a retrospective study, we could not access all the data required to determine the etiology in all cases. This may be due to patients being unable to recall the trauma, especially if unconscious or confused by their injuries. Initially, patients could struggle to remember the precise origin of the trauma, particularly if they were unconscious or disoriented due to the extent of their injuries. In many instances, the trauma may have been multifactorial, leaving patients confused about which incident precipitated the injury. In emergency settings, medical teams may prioritize quick care over comprehensive history-taking, resulting in insufficient documenting of the trauma etiology. Therefore, patients with unidentified etiologies were included in the study when other clinical records were complete. Conducting prospective studies will allow for more detailed collection and analysis of etiologies.

When cases of facial trauma are analyzed according to their etiologies, the gender ratio of the patients can differ from that of the overall ratio [9]. In the EURMAT project, men accounted for more than 80% of the patients with assault-related maxillofacial injuries in centers where the percentage of assault-related maxillofacial injuries was 40% or higher [7]. In our study, 29% of the patients had assault-related maxillofacial fractures, and men accounted for 90% of the patients with assault-related maxillofacial injuries. Although the overall male-to-female patient ratio was 3.9:1 in this study, the ratio was 9:1 for assault-related fractures. This high ratio (9:1) may have been related to the high rate of alcohol and drug use among adolescent and young men in our study population. Since our study was retrospective in nature, we could not obtain sufficient data on alcohol and drug use or investigate the relationship between alcohol and drug use and maxillofacial trauma. However, it is known that the rate of alcohol and drug use is high, especially among young men, in the region where our hospital is located. Prospective studies are needed to determine this relationship.

The literature indicates that maxillofacial fractures most commonly occur in individuals aged 20–30 years [5, 9, 22, 40]. In accordance with the literature, our results showed that the highest number of maxillofacial injuries occurred in the 19–28 years group, accounting for 23.9% of all cases. However, in the EURMAT project, most centers reported that the highest number of cases occurred in groups of patients with mean ages between 30 and 39 years, and this result was attributed to the increasing life expectancy and aging of European people [7].

In terms of the fracture site, in our study, nasal fractures were the most frequently observed, accounting for 63.7% (*n* = 1,384) of all cases. In contrast, previous studies have indicated that the mandible is the most common fracture site in maxillofacial injuries, frequently followed by the zygoma [7, 9, 40, 41]. The main reason for this difference is that all the studies that found that mandibular fractures were the most common maxillofacial fractures were conducted in oral and maxillofacial surgery departments, whereas most patients with nasal fractures are treated either in otolaryngology or plastic surgery departments. Among all the facial bones, the nasal bones are the most vulnerable to trauma due to their location. Therefore, it is not surprising that nasal fractures are among the most frequently reported fractures in cases of maxillofacial trauma and in studies of such trauma conducted in otorhinolaryngology, plastic surgery, and EDs [42]. Given that almost all patients with maxillofacial trauma first report to an ED, it is clear that studies conducted using data collected in EDs should be the most reliable in term of analyses of fracture locations. Arslan et al. conducted their study in an ED and found that the maxillary and nasal bones were the two most commonly fractured bones in patients with maxillofacial injury [42]. Regarding our results (i.e., nasal fractures were most common, followed by maxillary fractures), we are confident that they reflect the actual types of bones fractured in patients with maxillofacial injuries in the region where our hospital is located for two reasons. First, there is no oral and maxillofacial surgery department in our hospital or near our region. Second, regardless of whether surgical intervention is required or not, almost all patients with facial fractures are referred by the ED to our department. In the EURMAT project, which only included oral and maxillofacial departments, the most commonly fractured bone was found to be the mandible; however, the EURMAT collaborators also stated that because nasal fractures are often treated by otorhinolaryngology departments, the actual epidemiological features and percentages of facial fractures may be different [7].

When we examined the reported mandibular fractures, we found that condylar-subcondylar process fractures were the most commonly observed, accounting for 36.8%. These fractures were followed by symphysis/parasymphysis, corpus, coronoid, angle, and ramus fractures in decreasing numbers. Our findings were consistent with those of the EURMAT project, where the condyle was found to be the most fractured site of the mandible (34%) [7]. Previous studies showed that the most fractured part of the mandible was the corpus [40], symphysis/parasymphysis [43, 44], or angle [45]. Notably, even though mandibular fractures ranked fifth among the types of facial fractures in our study, they were the most common fracture type that required surgical intervention with GA.

The most common treatment method used across all cases was intervention with LA (*n* = 825, 47%). Specifically for nasal fractures, intervention with LA was also the most frequent treatment approach (*n* = 813, 58.3%). For mandibular fractures, surgical intervention under GA was the most commonly employed treatment (*n* = 64, 54.7%). Orbital fractures were primarily managed with conservative treatment and monitoring (*n* = 124, 63.3%). Similarly, maxillary fractures and frontal sinus anterior wall fractures were most frequently treated with conservative management (*n* = 149 [65.6%] and *n* = 32 [54.2%], respectively).

Surgical intervention was performed in 125 patients, with the most common procedure reduction under GA (*n* = 40, 28.8%). It was observed that the use of surgical treatment varied significantly according to age group, particularly for the intermaxillary fixation variable in the 0–7 years group. In this group, only one patient received a plate for mandibular fractures due to concerns about hindering bone development; the plate was removed early to prevent any potential negative effects on growth. Surgical interventions were most frequently performed on the first day (*n* = 42). The longest period before surgery was 20 days (*n* = 1). Postoperative complications developed in 10 patients. The most common complications were plate exposure (*n* = 6, 60%), wound-site infection (*n* = 3, 30%), and TMJ ankylosis (*n* = 1, 10%). These findings highlight the importance of considering the timing and type of surgical intervention, especially in younger patients, to avoid long-term developmental issues.

The present study has some limitations. The data, which were retrospectively collected from hospital records, did not include information about the patients’ alcohol or drug use. In addition, data on the etiology of the trauma were missing for some patients; thus, the etiology was not known in 20% of cases. Therefore, further prospective studies are needed to more precisely document the proportional differences between etiologies.

## Conclusion

The etiologies of maxillofacial fractures may differ among and within countries. In this study, assault was the leading cause of maxillofacial fractures, and men were four times more likely than women to experience maxillofacial trauma. The most affected age group was the 19–28 years group, and nasal fractures were the most frequently observed fracture type, accounting for 63.7% of cases.

Maxillofacial trauma is an avoidable injury, and formulating a systematic prevention strategy and carrying out life-long prevention that targets the high-risk factors of different age groups will decrease the burden of maxillofacial traumas.

## Data Availability

The data that support the findings of this study are available from the corresponding author upon reasonable request.

## Abbreviations

ED: Emergency Department
RTA: Road Traffic Accident
GA: General Anesthesia
LA: Local Anesthesia
CT: Computed Tomography
EUROMAT: European Maxillofacial Trauma
